# A Comprehensive Physiologically Based Pharmacokinetic Model of Nadolol in Adults with Renal Disease and Pediatrics with Supraventricular Tachycardia

**DOI:** 10.3390/ph17020265

**Published:** 2024-02-19

**Authors:** Samia Kalsoom, Muhammad Fawad Rasool, Imran Imran, Hamid Saeed, Tanveer Ahmad, Faleh Alqahtani

**Affiliations:** 1 Department of Pharmacy Practice, Faculty of Pharmacy, Bahauddin Zakariya University, Multan 60800, Pakistan; kalsoom088@gmail.com; 2 Department of Pharmacology, Faculty of Pharmacy, Bahauddin Zakariya University, Multan 60800, Pakistan; imran.ch@bzu.edu.pk; 3 Section of Pharmaceutics, University College of Pharmacy, Allama Iqbal Campus, University of the Punjab, Lahore 54000, Pakistan; hamid.pharmacy@pu.edu.pk; 4 Institute for Advanced Biosciences (IAB), CNRS UMR5309, INSERM U1209, Grenoble Alpes University, 38700 La Tronche, France; tanveer.ahmad@univ-grenoble-alpes.fr; 5 Department of Pharmacology and Toxicology, College of Pharmacy, King Saud University, Riyadh 11451, Saudi Arabia

**Keywords:** nadolol, physiologically based pharmacokinetic, PK-Sim, pharmacokinetic, renal impairment, pediatrics

## Abstract

Nadolol is a long-acting non-selective β–adrenergic antagonist that helps treat angina and hypertension. The current study aimed to develop and validate the physiologically based pharmacokinetic model (PBPK) of nadolol in healthy adults, renal-compromised, and pediatric populations. A comprehensive PBPK model was established by utilizing a PK-Sim simulator. After establishing and validating the model in healthy adults, pathophysiological changes i.e., blood flow, hematocrit, and GFR that occur in renal failure were incorporated in the developed model, and the drug exposure was assessed through Box plots. The pediatric model was also developed and evaluated by considering the renal maturation process. The validation of the models was carried out by visual predictive checks, calculating predicted to observed (R_pre/obs_) and the average fold error (AFE) of PK parameters i.e., the area under the concentration–time curve (AUC_0-t_), the maximum concentration in plasma (C_max_), and CL (clearance)_._ The presented PBPK model successfully simulates the nadolol PK in healthy adults, renal-impaired, and pediatric populations, as the R_pre/obs_ values of all PK parameters fall within the acceptable range. The established PBPK model can be useful in nadolol dose optimization in patients with renal failure and children with supraventricular tachycardia.

## 1. Introduction

Nadolol is a synthetic long-acting, non-selective β-adrenoceptor-blocker, devoid of direct myocardial depression and lacks membrane-stabilizing activity [1,2,3]. Nadolol was released in 1979 in the United States for clinical use and later was approved by the Food and Drug Administration (FDA), for the management of angina pectoris and hypertension [4,5]. It competitively blocks the cardiac β–1 receptor, which causes a decrease in peripheral vascular resistance and leads to a reduction in systolic and diastolic blood pressure [3]. Non-FDA-approved indications of nadolol are atrial fibrillation, liver cirrhosis, thyrotoxicosis, and supraventricular tachycardia [4]. Nadolol is classified in the Biopharmaceutics Classification System (BCS) class 3, with low permeability and high solubility [6,7]. It is excreted in the milk of lactating mothers [8] and considered in category C medication in pregnancy [9]. Nadolol has a long elimination half-life (t_½_) ranging from 14 to 24 h [10]. It is mainly bound to alpha–1 acid glycoprotein up to 27% [11]. Nadolol does not undergo hepatic metabolism and 75% of the administered dose is eliminated through kidneys in a non-metabolized form and the remaining drug is cleared from the body by non-renal (biliary) pathways [5,10,12].

The clinical efficacy of hypertensive drugs (β-blockers) depends upon the better understanding of the pharmacokinetic properties and their relation with blood-lowering action [13]. Nadolol’s therapeutic serum concentration is within the range 0.01–0.25 mL/L [14,15], its reported Emax is 68.09 ± 12.81 and ED50 is 126.62 ng min^−1^ [16,17]. Nadolol and other B-blockers are highly effective in the prevention of potentially lethal arrhythmia in 50% of patients; this response is comparable to class IA and class IB anti-arrhythmic agents [18]. β-blockers have a low incidence of organ toxicity such as hepatic damage and agranulocytosis, which are of great concern in class I and class III anti-arrhythmic agents [18]. In patients with renal failure, the half-life of nadolol becomes prolonged by up to 45 h [19] and as a result the drug exposure is increased in the body and there is a need for dose reduction in such patients as nadolol is cleared 95% from the kidneys [17].

Consequently, pathological conditions such as chronic kidney disease (CKD) may alter the disposition and exposure of nadolol, which causes a modification in therapeutic efficacy or adverse effects. The serum t_½_ of nadolol becomes prolonged from 18 to 68.6 h in proportion to the kidney functioning from moderate to severe renal impairment [20]. Therefore, according to the creatinine clearance, a suitable dosage regime should be made in patients with renal impairment receiving nadolol [20]. In this regard, whole-body physiologically based pharmacokinetic (PBPK) modeling and simulation can incorporate the pathophysiological and biochemical changes of liver cirrhosis and renal impairment and the assessment of these effects on drug exposure and disposition [21,22,23]. The PBPK modeling and simulations can also help estimate the effect of intrinsic and extrinsic factors on the drug’s PK parameters that are associated with clinically relevant endpoints [24,25].

The image of PBPK modeling was first introduced in 1937 by Teorell [26]. Recent regulations from the regulatory bodies i.e., the European Medicines Agency (EMA) and FDA, highlight the significance and need for PBPK modeling and simulation reports to build on the improved pattern of pediatric drug development studies for minimizing the off-label usage of drugs in diseased pediatric subjects [25,27]. Nearly 50% of all the medications administered to infants and children have not been examined in this population and approximately 90% of drugs are used off-label in pediatrics of age less than one year which leads to an increased number of adverse drug reactions [28,29]. In this respect, PBPK modeling and simulation are ideal for analyzing the PK changes between children and adults and making child-appropriate-dosing and applying safety precautions [30]. There are pronounced nadolol PK changes that may take place when it is used as off–label in infants and children for the treatment of supraventricular tachycardia [31]. The developed PBPK models can forecast age-related changes and incorporate these modifications into the models that help predict the safe dose and drug exposure in pediatrics [30]. Previously, PBPK models of many drugs have already been published in the literature for renal failure [32], liver cirrhosis [33], and special populations [34,35].

To the best of our knowledge, this is the first PBPK report of nadolol in humans. The rationale for carrying out the recent study was to build and validate the whole-body PBPK drug–disease model of nadolol in pediatrics and patients suffering from chronic kidney disease. The ultimate goal of conducting the present study is to analyze the impact of renal failure on nadolol exposure and to address the effect of age of the pediatric population on nadolol disposition to generate a safe and effective dosage regime in CKD and pediatric populations where no clinical data are available.

## 2. Results

### 2.1. Healthy Adults after Intravenous Application

**Figure 1:**
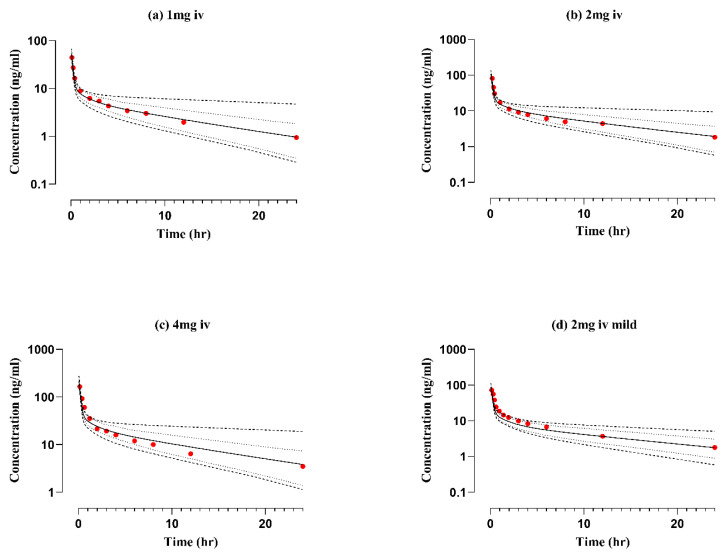
Predicted and observed visual predictive checks of concentration–time profiles of nadolol in “healthy subjects” after intravenous nadolol administration (**a**–**c**) [36] and (**d**) [2]. Observed data (red solid circle), arithmetic mean (solid line), minimum and maximum values (dashed lines), 5th and 95th percentiles (dotted lines), iv (intravenous).

An initial full-body PBPK model was built in healthy adults after IV nadolol administration with doses ranging from 1 to 4 mg. The visual predictive checks illustrated in Figure 1 show that the developed PBPK model successfully predicted the PK profiles of nadolol after IV administration. The observed data were predominantly ranging between the 5th and 95th percentile range of the simulated PK data shown in Table 1. The predicted-to-observed ratio of AUC_0-t_, C_max_ ranged from 0.71 ng.h/mL to 1.02 ng.h/mL and 1.16 ng/mL to 1.28 ng/mL, respectively. All the PK parameters CL, AUC_0-t_ and C_max_ were within the twofold error range as given in Table 1 and Table 2.

### 2.2. Healthy Adults after Oral Application

**Figure 2:**
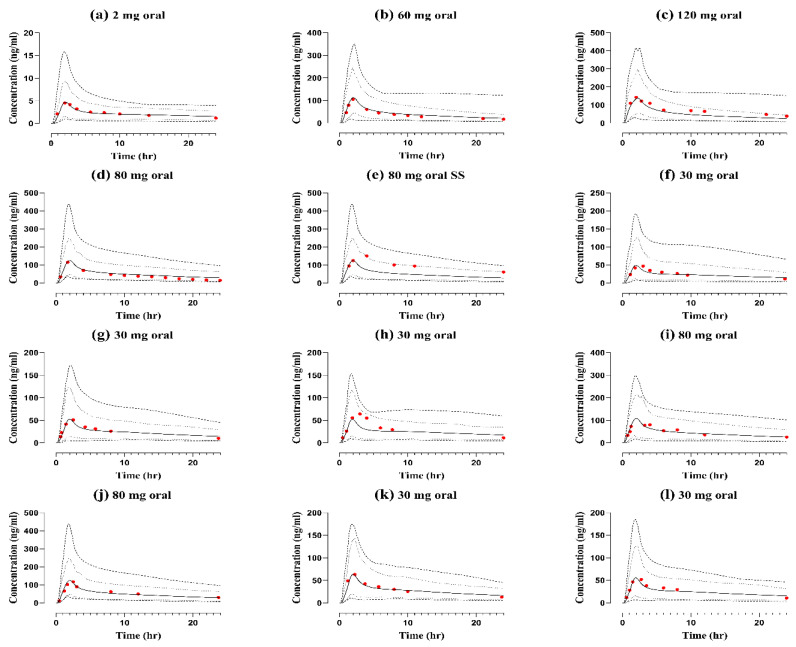
Predicted and observed visual predictive checks of concentration–time profiles of nadolol in “healthy subjects” after oral dose administration (**a**) 2 mg [2] (**b**) 60 mg [37] (**c**) 120 mg [37] (**d**) 80 mg [38] (**e**) 80 mg [38] (**f**) 30 mg [39] (**g**) 30 mg [40] (**h**) 30 mg [41] (**i**) 80 mg [42] (**j**) 80 mg [43] (**k**) 30 mg [44] (**l**) 30 mg [45]. Observed data (red solid circle), arithmetic mean (solid line), minimum and maximum values (dashed lines), 5th and 95th percentiles (dotted lines), SS (steady-state plasma concentration).

The developed nadolol compound file was evaluated using reported PK data from 10 clinical studies in healthy subjects after oral nadolol administration. Figure 2 shows the comparison of simulated and observed mean concentration–time profiles with doses ranging from 30 to 120 mg. The visual predictive checks show that all the observed data sets are predominantly within the 5th to 95th percentiles, minimum and maximum limits of the predicted PK data, qualifying the visual verification. The R_pre/obs_ of PK parameters i.e., AUC_0-t_ ranged from 0.60–1.18, C_max_ between 0.81 and 1.35 and CL ranged from 0.61 to 1.25, and all were in the acceptable range of twofold as summarized in Table 1. The values for the average fold error of all the simulated doses for model evaluation are given in Table 2.

### 2.3. Renal Failure Population

**Figure 3:**
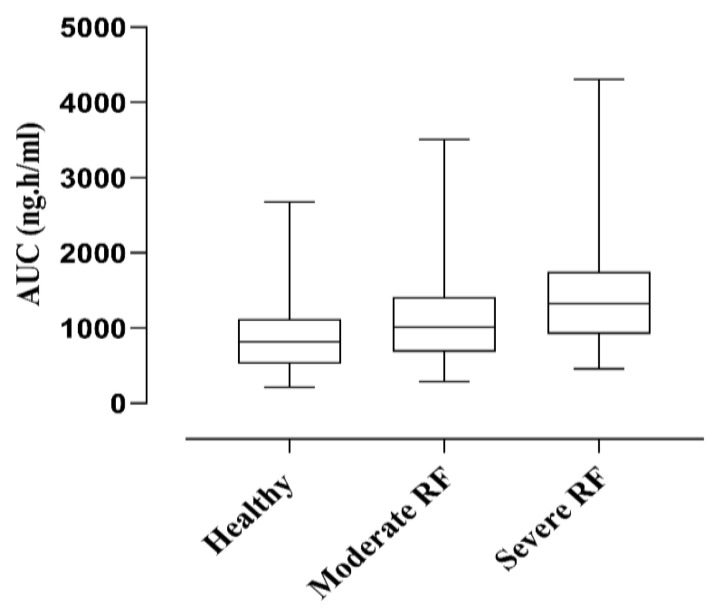
Box plots for comparison of simulated plasma AUC, after oral 80 mg nadolol to healthy adults, moderate and severe renal failure patients. Box and Whisker plots represent as middle band (median), lower and upper quartiles (25th and 75th percentiles), minimum and maximum values in the data set (lower and upper whiskers). AUC area under the concentration–time curve from time zero to “t”, RF = renal failure.

It is reported in a study that the serum concentration of nadolol increased from 50.4 to 440.3 ng/mL in patients with moderate to severe kidney failure [20]. Box plots were generated, and the AUC was compared, for dose optimization in renal failure patients. The mean AUC in healthy individuals 916.7 ng.h/mL was increased to 1098 ng.h/mL in moderate RF and 1394 ng.h/mL in patients with severe RF. This greater increase in AUC with increased severity of renal impairment can be visualized and the necessary dose adjustments can be made as shown in Figure 3.

### 2.4. Pediatric Population

**Figure 4:**
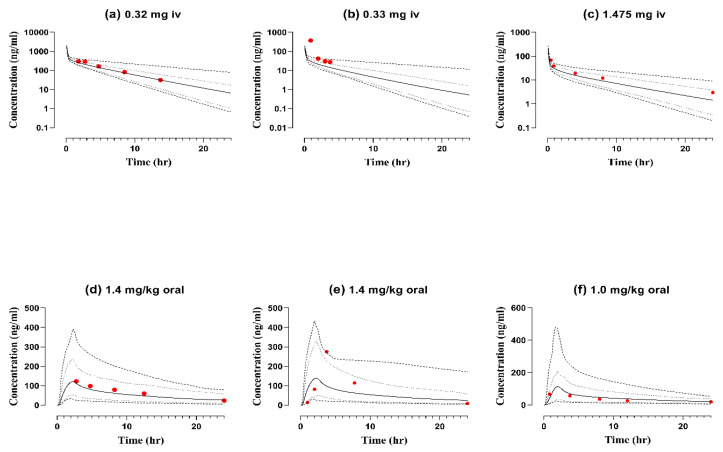
Visual predictive checks of observed and simulated data sets both after intravenous (**a**–**c**) and oral (**d**–**f**) nadolol administration to infants and children with supraventricular tachycardia [31]. Observed data (solid red circle), arithmetic mean (solid line), minimum and maximum values (dashed lines), 5th and 95th percentiles (dotted lines), iv = intravenous.

In pediatrics with ages ranging from 3 months to 14 years, the observed and simulated concentration times were compared both after IV and oral administration (Figure 4). The calculated predicted-to-observed ratios R_pre_/_obs_ of AUC after IV dose administration ranging from 0.98 ng.h/mL to 0.72 ng.h/mL and C_max_ was 1.98 ng/mL to 0.57 ng/mL, whereas after oral administration AUC values ranged from 0.51 ng.h/mL to 1.26 ng.h/mL and values of C_max_ were between 1.68 ng/mL and 1.00 ng/mL. The R_pre_/_obs_ ratios for the predicted and observed data sets were within the limits of a twofold error range except for the AUC and C_max_ in one population category of pediatrics after IV administration, summarized in Table 1. The developed nadolol pediatric model is further evaluated by calculating the AFE for PK parameters C_max_, CL, and the AUC given in Table 2.

### 2.5. Age-Related Changes in Exposure

**Figure 5:**
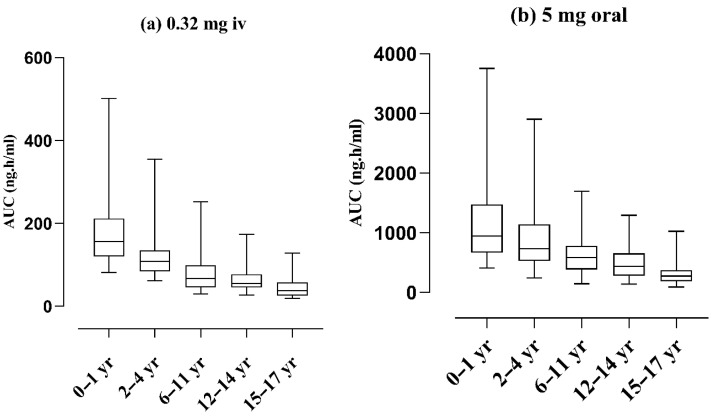
Box plots for simulated nadolol plasma AUC, (**a**) intravenous (**b**) oral at different stages of child development the age between 0 and 1 year classified as infants; age between 2 and 4 years as toddlers; age ranges 6–11 years come into middle childhood; age ranges 12–14 years fall into the young teen category; ages from 15 to 17 years come under the teenager category. Box and Whisker plots represent the 50th percentile or median (middle band), 25th and 75th percentiles (lower and upper quartiles), lower and upper whisker (minimum and maximum value in the data set). AUC area under the curve.

All the categories of child development made by the CDC child development age chart [46] were visualized for AUC comparison with the help of Box plots after the same IV and oral doses. A steeper decrease in AUC can be seen both after IV and oral dose administration as the clearance increases from infants to teenagers, as shown in Figure 5. The mean AUC after the IV dose 0.32 mg administration was decreased from 166.8 ng.h/mL to 42.35 ng.h/mL and after the oral dose 5 mg administration was decreased from 1149.3 ng.h/mL to 300.4 ng.h/mL from infants to teenagers.

## 3. Discussion

In this current study, a comprehensive nadolol PBPK model was developed and validated thoroughly for systematically assessing the impact of changes in various physiological parameters on nadolol PK profiles. The PBPK model was established and evaluated by utilizing clinical PK studies in adult healthy volunteers, patients with impaired kidney function, and pediatric patients. The process of the model development was initiated by simulating the clinical PK profiles in healthy subjects after IV drug administration. Once the IV adult healthy nadolol model was built and evaluated, absorption parameters were incorporated, and oral PK data were simulated and validated. The predicted AUC and C_max_ in healthy populations both after IV and oral administration were comparable to observed PK data as perceived by R_pre/obs_ values (Table 1). A nadolol-diseased PBPK model was successfully established in patients with renal failure and pediatrics with supraventricular tachycardia.

Nadolol is predominantly 75% excreted through the renal filtration process and hepatic metabolism does not contribute to its clearance [2]. The predicted plasma AUC of nadolol increased from 1098 ng.h/mL to 1394 ng.h/mL in patients with moderate to severe renal failure, similar to the clinical study observations in renally impaired patients [20]. In this study, the developed PBPK model in renal failure showed that the decline in normal kidney functioning leads to a decrease in GFR resulting in an increase in the AUC. This is because the drug remains in the body for longer periods, nearly 68 h, even after a single administration of oral 80 mg nadolol [20]. This point is further confirmed through another study in which the oral 40 mg of nadolol is administered to patients with various degrees of kidney failure [47]. As we increase the dose of nadolol, drug clearance will become compromised in patients with renal failure, which can lead to drug accumulation; therefore, there is a need to make dose adjustments in patients with renal failure.

The established PBPK model in this current study can be useful for making dose adjustments according to the degree of renal failure. Moreover, there are multiple physiological and biochemical changes that occur in the various degrees of renal impairment, such as the protein binding changes, GFR, hematocrit, gastric emptying time and cytochrome (CYP) P450 enzyme abundances, fraction unbound in plasma, small intestinal transit time reported in the studies [21,23]. The values of these physiological parameters can be incorporated into the simulator program for simulation of the renal failure patient populations and making dose optimizations accordingly [21,23]. The PBPK model of renal impairment in this report was also built by incorporating the physiological changes reported previously [23].

The nadolol–pediatric PBPK model is developed and evaluated for a clinical study in which nadolol is used as an off-label for treating supraventricular tachycardia in infants and children [31]. This PBPK pediatric model successfully presented the fact that the simulated IV and oral concentration–time curves were comparable to the observed PK data sets as R_pre/obs_ ratios were within the 1.5-fold error range, except for the one IV virtual profiles where it was underpredicted (Figure 5). This underprediction occurred because of the difference in the dosing schedule adopted in the respective clinical studies, as two IV doses were given to patients at times t = 0 and t = 45 h, while the administration protocol for all other patients in this study was once daily [31].

Conventionally, pediatric doses are calculated based on the adult doses, after making adjustments by taking into account the standardized body weight (70 kg) or the body surface area (BSA) [48]. But in many scenarios, infants and children do not show the PK of “mini adults” due to the physiological differences and expression of enzymes as a function of age in the pediatric age group. The maturational changes that occur during the developmental stages of pediatrics as organ blood, hepatic blood flow, height, and weight change in proportion to age have been reported previously [30]. In this present study, IV and the oral fixed-dose effect were accessed by making AUC Box plots of each pediatric population category given above. As age increases, the AUC declines because the maturational changes occur, i.e., GFR, hematocrit, gastric emptying time, and organ blood flow. This confirms the need to carefully monitor the dose regime in pediatric populations.

The present work’s strength is that for the first time a PBPK Nadolol model has been reported, which successfully predicted the PK in healthy, diseased and pediatric populations following IV and oral administration. The presented PBPK model may be helpful in suggesting individualized dosage regimen in adults with varying degrees of renal failure. Since nadolol clearance shows a steeper change from the infant to the teenager age group, the developed model can be very useful in predicting its dosage in children of different age groups. Regulatory bodies are trying to increase the focus on apprehending the drug PK in the pediatric population, but this population has until now been under-represented in the published literature, and this limits the model evaluation in this population. The presented pediatric model was only evaluated with one reported clinical PK study and this can be treated as a limitation of this work. Moreover, due to the availability of limited published literature in the pediatric population, there is always an uncertainty regarding the model input parameters. Most of the clinical data used for model evaluation were extracted by digitizing the publication plots and minor errors cannot be completely ruled out. The model input value of the specific intestinal permeability was optimized for improving the visual predictive checks and comparison of PK parameters.

## 4. Methods

### 4.1. Modeling Software

A whole-body PBPK model was built by employing the free-to-use modeling software PK-Sim^®^ of version 9.1 (Open Systems Pharmacology Suite, Bayer Technology Services, Leverkusen, Germany https://www.open-systems-pharmacology.org/ (accessed on 12 July 2021)). The parameterization and optimization of model input parameters were performed within a PK-Sim^®^ program.

### 4.2. PBPK Modeling Strategy

The procedure of PBPK model development began with an extensive literature search to obtain the input physico-chemical parameters and data on absorption, distribution, metabolism, and elimination, as well as relevant published clinical studies of nadolol. Then, these physico-chemical compound parameters were incorporated into the modeling simulator for simulating and evaluating the intravenous (IV) PK data in adult healthy individuals. Subsequent to the successful development of a IV adult healthy model, oral PK profiles of healthy individuals were simulated, and the observed data were superimposed onto these simulated concentration–time curves for assessing the model predictions. The drug–disease model in CKD patients and the pediatric population was built by incorporating the physiological alterations.

### 4.3. Building Blocks for PBPK Model Development

The physicochemical properties of nadolol such as pKa, octanol-water coefficient (LogP), solubility and molecular weight are obtained from a thorough search of the published literature. Specific intestinal permeability (P_eff, man_) of nadolol for the absorption of oral drug administration was optimized to 1.03 × 10^−6^ cm/min from the calculated value 6.4 × 10^−7^ cm/min of the modeling program with the help of visual predictive checks. The reported value of P_eff, man_ ranges from 0.018 × 10^−5^ cm/s to 0.13 × 10^−6^ cm/s [49,50]. For the distribution estimation, the “PK-Sim standard” method of distribution was used. Table 3 summarizes all the drug-specific input elements used for the model building.

### 4.4. Clinical Pharmacokinetic Data

An exhaustive search was conducted for all the relevant PK data profiles required for building the PBPK drug–disease model of nadolol. The clinical studies were sorted based on the published PK data profiles in the healthy, diseased and special populations (i.e., pediatrics) in which nadolol is administered by either IV or the oral route. From the selected studies, concentration–time curves were digitized using GetData Graph Digitizer software version 2.26.0.20.

A total of 14 PK studies were included for the model development, in which 12 studies (2 IV, 10 oral) were in healthy individuals, 2 were in diseased populations, 1 was in CKD patients, and another study was in pediatrics with supraventricular tachycardia. One-third of the selected studies were used for the PBPK model building and the remaining two-third were utilized for model validation. Nadolol PK profiles in healthy adults after oral administration were available in higher numbers, providing a rich data set for model verification. Details on the population demographics of the included studies are given in Table 4.

### 4.5. Renal Impairment Population

Nadolol PK was simulated in 100 virtual subjects in three different virtual populations, with ages ranging from 18 to 74; the proportion of females (25%) and a dosing schedule of 80 mg orally once daily were designed as reported in the clinical study [20], while the height and weight were set as the default setting of the PK-Sim simulator. The categories of virtual populations which were used for simulations included “Healthy volunteers” as a template file, “Moderate RF” (GFR between 30 and 60) and “Severe RF” (GFR < 30) made in the PK-Sim simulator, although the “RF populations” account for physiological changes in the hematocrit, glomerular filtration rate (GFR), and gastric emptying time [23]. The nadolol-renal failure model was developed by calculating and incorporating the respective changes for moderate and severe renal failure i.e., hematocrit (0.42, 0.39), gastric emptying time (20.4 min, 24.6 min) and GFR (45 mL/min, 18 mL/min).

### 4.6. Pediatric Model

**Figure 6:**
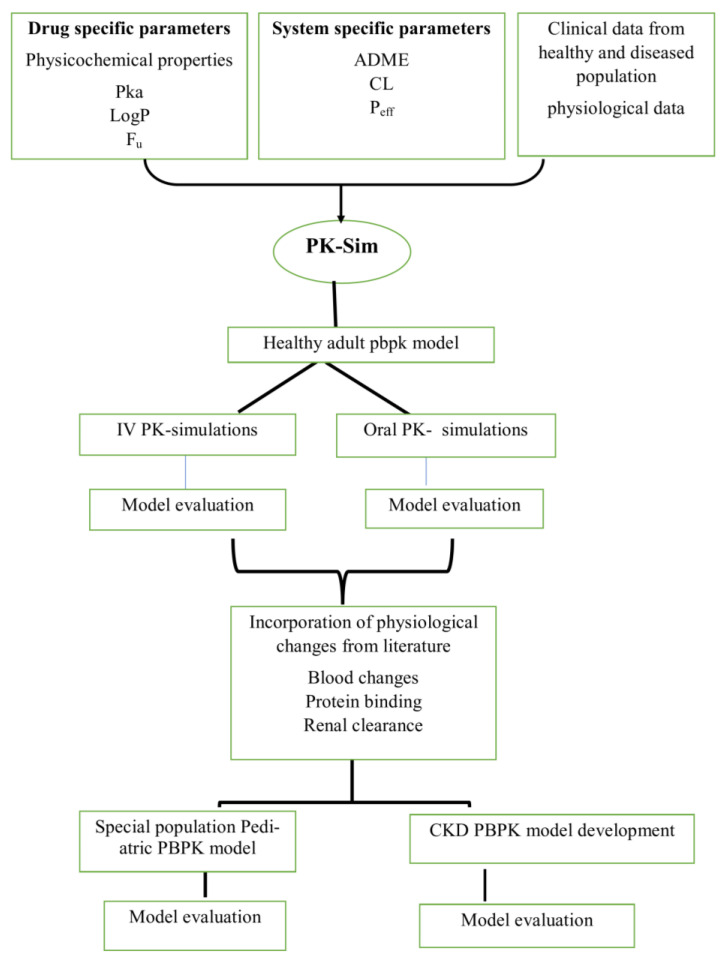
Workflow of nadolol PBPK model. LogP_o:w_ = Octanol–water partition coefficient, pKa = Acid dissociation constant, ADME = Absorption, Distribution, Metabolism, Excretion, P_eff_ = Specific intestinal permeability, IV = intravenous, PK = Pharmacokinetic, CKD = chronic kidney disease.

The infants and children with supraventricular tachycardia aged between 1 month and 14 years, after both IV and oral nadolol administration, were used for building a nadolol–pediatric model. Three categories of pediatrics virtual populations were made as “3–months”, “5–months” and “121–months” in the PK-Sim simulator, as provided in the selected clinical study [31]. Additionally, to analyze the fixed-dose effect after IV (0.32 mg) and oral (5 mg) administration on different age groups of child development, Box–Whisker plots were used. The categories of different virtual populations “Infants aged between 0–1 year”, “Toddlers aged ranging 2–4 years”, “Middle Childhood from 6–11 years”, “Young teens ranging from 12–14 years” and “Teenagers between 15–17 years” were generated in accordance with the Centers for Disease Control and Prevention (CDC) child development age chart [46]. The overall PBPK model developing strategy workflow is presented in Figure 6.

### 4.7. Model Verification

For model evaluation, a virtual population comprising 100 individuals was selected and simulated based on the observed population characteristics and administration protocol for each PK study illustrated in Table 4. Initially, the PBPK-developed model was evaluated with visual predictive checks by superimposing the observed data on the simulated PK curves. The predicted data are the arithmetic mean, maximum and minimum, 5th percentiles and 95th percentiles. Non-compartmental analysis (NCA) for PK parameter analyses was performed by using the Microsoft Excel add-in program PKSolver [55]. Observed and predicted values for specific PK parameters including C_max_ (maximum plasma concentration), AUC_0-t_ (area under the concentration–time curve), and Cl (clearance) were evaluated. Results were compared using the ratio of predicted/observed PK parameters and average fold error values (AFE), calculated by using the equation given below. The model evaluation criteria were considered fulfilled when the predicted PK parameters (AUC, C_max_, CL) fell within a twofold error (0.5–2) of the observed PK parameters [56,57].

Equations for calculating the R_pre_/R_obs_ and AFE are given below:

(1) $$R=\frac{predicted\;value\;of\;PK\;parameter}{observed\;value\;of\;PK\;parameter}$$

Average-fold error (AFE)

(2) $$AFE=10^{\frac{\sum \log\left(fold\;error\right)}{N}}$$

where N represents the number of samples and the fold-error is the predicted/observed parameter estimates ratio.

## 5. Conclusions

In the presented report, we successfully established and evaluated the PBPK model of nadolol in the adult healthy population, after IV and oral dose administration, in adult patients with renal impairment and pediatric patients. The developed pharmacokinetic PBPK model can serve as a useful tool for supporting dosing optimization in pediatric and adult patient populations with diseased states such as differing degrees of renal failure and in pediatric populations undergoing renal function maturation.

## Figures and Tables

**Table 1:** Predicted-to-observed ratios in healthy and diseased populations after IV and oral nadolol administration.

Sr. No	Dose (mg)	C_max_ (ng/mL)		Ratio	AUC_0-t_ (ng.h/mL)		Ratio	CL (L/h)		Ratio	Ref.
		Predicted	Observed		Predicted	Observed		Predicted	Observed		
IV Healthy											
	1	52	44.6	1.16	78	82	0.95	10.5	10.6	0.99	[36]
2.	2	104	81	1.28	157	172	0.91	19.3	20.4	0.94	[36]
3.	4	209	165	1.26	314	306	1.02	46.2	44.4	1.04	[36]
4.	2	86	71	1.19	128	179	0.71	13.5	14.1	0.96	[2]
Oral Healthy											
	2	4.7	4.4	1.07	71.9	68.6	1.04	40	56	0.71	[2]
2.	60	112	105	1.06	973.5	972.7	1.00	2280	2820	0.80	[37]
3.	120	138.4	143.3	0.96	1215	1992.5	0.60	7524	6240	1.20	[37]
4.	80	125.2	115	1.08	1202.9	1011.3	1.18	3300	5360	0.61	[38]
5.	80 (SS)	125	151	0.82	1205	2224	0.54	1126	1440	0.78	[38]
6.	30	125.7	117.8	1.05	1583	1830.7	0.79	1050	1020	1.02	[39]
7.	30	53	51	1.03	722	716	1.00	1080	1140	0.94	[40]
8.	30	52	64	0.81	802	838	0.95	930	960	0.96	[41]
9.	80	108	80	1.35	1063	1071	0.99	3520	3840	0.91	[42]
10.	80	125	117	1.07	1583	1813	0.87	3824	3040	1.25	[43]
11.	30	64.4	63.2	1.01	830	840.4	0.98	960	990	0.96	[44]
12.	30	55	52	1.05	759.7	765.5	0.99	1050	1020	1.02	[45]
IV Pediatrics Population											
	0.32	360	346	1.04	2255	2288	0.98	0.22	0.20	1.13	[31]
2.	0.33	191	1312	0.14	163	351	0.46	0.07	2.7	0.25	[31]
3.	1.475	198	99.5	1.98	244	337	0.72	57.5	47.2	1.21	[31]
Oral Pediatrics Population											
	1.4 ^∞^	122	122.4	1.00	1281	1479.3	0.86	1.09	0.99	1.10	[31]
2.	1.4 ^∞^	1306	2148	0.60	140	275	0.51	1.82	3.40	0.53	[31]
3.	1.0 ^∞^	113	66.8	1.68	1013.6	799	1.26	0.66	0.87	0.75	[31]

IV = intravenous, C_max_ = maximum concentration, AUC_0-t_ = area under the concentration–time curve from time zero to “t”, CL = clearance, SS = steady state, ^∞^ mg/kg.

**Table 2:** Calculated average fold error (AFE) in healthy and diseased populations after IV and oral administration of nadolol.

PK Parameters	AFE (Average Fold Error)
IV Healthy	
Cmax	0.87
AUC	0.56
Cl	0.98
Oral Healthy	
Cmax	1.06
AUC	0.99
Cl	0.76
IV Pediatrics	
Cmax	1.10
AUC	0.98
Cl	0.85
Oral Pediatrics	
Cmax	1.03
AUC	0.76
Cl	0.86

C_max_ = maximum serum concentration, AUC = area under the curve from time zero to t, Cl = clearance.

**Table 3:** Summary of nadolol-specific input parameters for model development.

Input Parameters	Value/Model	References
Physico-chemical Characteristics		
Molecular Weight (g/mol)	309.4	[51]
Water Solubility (mg/mL)	8.33	[49]
pKa	9.17	[7]
LogP o:w	0.81	[52]
Absorption		
P_eff, man_ (cm/min)	1.03 × 10^−6^	Optimized by Visual Predictive Checks
Distribution		
f_u_	0.7	[53]
Distribution Prediction Method	PK-Sim Standard	
Excretion		
CL_R_ (ml/min)	131	[36]
CL_T_ (ml/min)	219	[36]

pKa = acid dissociation constant, LogP_o:w_ = octanol–water partition coefficient, P_eff, man_ = effective specific permeability, f_u_ = unbound fraction of drug, CL_R_ = renal clearance, CL_T_ = total clearance.

**Table 4:** Population characteristics for nadolol pharmacokinetics in healthy, diseased and pediatric subjects.

Sr. No	Population	No. of Participants	Dose (mg)	Route	Females Proportion	Age (Years)	Weight (kg) Ranges	References
	Healthy	9	1, 2, 4	IV ᵜ	0	20–27	63.6–96.6	[36]
2.	Mild hypertensive	4	2	IV ᵜ	0	43–54	76–111	[2]
				Oral				
3.	Healthy	8	80	Oral	0	21–24	73–84	[38]
4.	Healthy	7	60, 120	Oral	4	24–42	42–70	[37]
5.	Healthy	11	30	Oral	0	21–29	47–98.6	[39]
6.	Healthy	8	30	Oral	2	20–30	47.5–57.7	[40]
7.	Healthy	12	30	Oral	6	20–63	48–98.9	[41]
8.	Healthy	7	80	Oral	0	19–22	N/R *	[42]
9.	Healthy	8	80	Oral	4	18–23	51.8–74.9	[43]
10.	Healthy	8	30	Oral	0	N/R *	N/R *	[44]
11.	Healthy	12	80	Oral	0	18–30	34–76	[54]
12.	Healthy	13	30	Oral	7	21–63	51.3–88.5	[45]
13.	CKD ^ⱷ^	24	80	Oral	11	22–74	N/R *	[20]
14.	Pediatrics	6	0.05–5 ^∞^	IV ᵜ	1	3 months–14 years	6.4–50	[31]
			0.5–2 ^∞^	Oral				

ᵜ Intravenous, * Not reported, ^ⱷ^ Chronic kidney disease, ^∞^ mg/kg.

## Data Availability

The original contributions presented in the study is included in the article, further inquiries can be directed to the corresponding author/s.
